# gamma‐secretase modulation: from bench to clinic

**DOI:** 10.1002/alz.090509

**Published:** 2025-01-09

**Authors:** Kevin Rynearson

**Affiliations:** ^1^ UCSD, San Diego, CA USA

## Abstract

Cerebral beta‐amyloid accumulation is the key initiator of Alzheimer’s disease (AD) pathology. Most familial early‐onset AD mutations in the *APP, PSEN1/2* genes increase the ratio of Abeta42:Abeta40, which drives beta‐amyloid accumulation in the brain. In 2001, the late Steve Wagner, Maria Kounnas, and I directed an agnostic high‐throughput screen for compounds that would reverse the Abeta42:Abeta40, ratio, and discovered the first non‐NSAID (second generation) gamma secretase modulators (GSM) at TorreyPines Therapeutics. Since then, in academia, we designed several novel structures similar to, but distinct from our original GSMs with predicted higher aqueous solubility. These compounds were then developed in the Wagner and Tanzi labs at UCSD and MGH, respectively with funding from the NIH and Cure Alzheimer’s Fund. Importantly, unlike gamma secretase inhibitors, our GSMs do not inhibit γ‐secretase activity, i.e. do not significantly inhibit NOTCH cleavage (up to 25 uM), but, instead, allosterically modulate gamma secretase activity, reducing generation of Aβ42 and to a lesser extent Aβ40, while enhancing production of Aβ37 and Aβ38, with IC50’s in the low nanomolar range. The preclinical development of our GSM’s evolved into four distinct chemical classes and involved testing over 1000 analogs, leading to our main clinical candidate, GSM776890, for which an IND has been submitted to the FDA (sponsored by Acta Pharmaceuticals, Boston). GSM776890 exhibits robust time‐ and dose‐dependent efficacy in both acute and chronic studies across various species, including a transgenic AD mouse model. GSM776890 displayed a >40‐fold safety margin in rats, with no observed adverse effects to the 50% effective AUC or AUC effective, which is needed to reduce Aβ42 levels by 50% in rat brain (Rynearson et al. JEM, 2021). Given the recent success of Abeta immunotherapy in clinical trials, GSM776890 represents an oral molecule alternate to managing beta‐amyloid levels as means for preventing and treating AD.

